# Data standards can boost metabolomics research, and if there is a will, there is a way

**DOI:** 10.1007/s11306-015-0879-3

**Published:** 2015-11-17

**Authors:** Philippe Rocca-Serra, Reza M. Salek, Masanori Arita, Elon Correa, Saravanan Dayalan, Alejandra Gonzalez-Beltran, Tim Ebbels, Royston Goodacre, Janna Hastings, Kenneth Haug, Albert Koulman, Macha Nikolski, Matej Oresic, Susanna-Assunta Sansone, Daniel Schober, James Smith, Christoph Steinbeck, Mark R. Viant, Steffen Neumann

**Affiliations:** Oxford e-Research Centre, University of Oxford, 7 Keble Road, Oxford, OX1 3QG UK; European Molecular Biology Laboratory, European Bioinformatics Institute (EMBL-EBI), Wellcome Trust Genome Campus, Hinxton, Cambridge, CB10 1SD UK; National Institute of Genetics, Mishima, Shizuoka 411-8540 Japan; RIKEN Center for Sustainable Resource Science, Yokohama, Kanagawa 230-0045 Japan; University of Manchester, Centre for Endocrinology and Diabetes, Old St Mary’s Building, Hathersage Road, Manchester, M13 9WL UK; School of Chemistry, Manchester Institute of Biotechnology, The University of Manchester, 131 Princess Street, Manchester, M1 7DN UK; Metabolomics Australia, The University of Melbourne, Parkville, VIC 3010 Australia; Computational and Systems Medicine, Department of Surgery and Cancer, Imperial College London, South Kensington, London, SW7 2AZ UK; MRC Human Nutrition Research, Elsie Widdowson Laboratory, 120 Fulbourn Road, Cambridge, CB1 9NL UK; Bordeaux Bioinformatics Center, Université de Bordeaux, Bordeaux, France; CNRS/LaBRI, Université de Bordeaux, Talence, France; Steno Diabetes Center, 2820 Gentofte, Denmark; Department of Stress and Developmental Biology, Leibniz Institute of Plant Biochemistry, Weinberg 3, 06120 Halle, Germany; School of Biosciences, University of Birmingham, Edgbaston, Birmingham, B15 2TT UK; Department of Applied Mathematics and Theoretical Physics, Cambridge Computational Biology Institute, University of Cambridge, Wilberforce Road, Cambridge, CB3 0WA UK

**Keywords:** Metabolomics, Data standards, Mass spectrometry, NMR, Experimental metadata, Data sharing

## Abstract

Thousands of articles using metabolomics approaches are published every year. With the increasing amounts of data being produced, mere description of investigations as text in manuscripts is not sufficient to enable re-use anymore: the underlying data needs to be published together with the findings in the literature to maximise the benefit from public and private expenditure and to take advantage of an enormous opportunity to improve scientific reproducibility in metabolomics and cognate disciplines. Reporting recommendations in metabolomics started to emerge about a decade ago and were mostly concerned with inventories of the information that had to be reported in the literature for consistency. In recent years, metabolomics data standards have developed extensively, to include the primary research data, derived results and the experimental description and importantly the metadata in a machine-readable way. This includes vendor independent data standards such as mzML for mass spectrometry and nmrML for NMR raw data that have both enabled the development of advanced data processing algorithms by the scientific community. Standards such as ISA-Tab cover essential metadata, including the experimental design, the applied protocols, association between samples, data files and the experimental factors for further statistical analysis. Altogether, they pave the way for both reproducible research and data reuse, including meta-analyses. Further incentives to prepare standards compliant data sets include new opportunities to publish data sets, but also require a little “arm twisting” in the author guidelines of scientific journals to submit the data sets to public repositories such as the NIH Metabolomics Workbench or MetaboLights at EMBL-EBI. In the present article, we look at standards for data sharing, investigate their impact in metabolomics and give suggestions to improve their adoption.

## Introduction

Data standardisation efforts can trigger ambivalent and often polarised reactions. Already when reading the normal scientific literature, experiments are described in a rather heterogeneous way with different levels of detail, or ambiguous and sometimes underspecified concepts such as “replicate”, where the true meaning is often buried in traditions specific to human/plant or bacterial research disciplines. With biological assays increasingly represented in digital form, biology has become a data-intensive field of disparate methods, with images, sequence reads and spectra, to name only a few, all being acquired by the droves. Modern scientists and data managers are therefore faced with the tremendous challenge of handling, preserving and archiving large amounts of data.

Metabolomics is no exception: PubMed returns 2460 hits for the search terms “metabolomics or metabonomics” from the year 2014 alone. Yet, only a tiny fraction of the data from this scientific output has been made available to the scientific community, data-miners and so-called data-wranglers through public repositories. In recent years, the notion of FAIR (**F**indable, **A**ccessible, **I**nteroperable and **R**eusable) research data objects has been endorsed by an increasing number of researchers and organisations, including the Dutch Techcenter for Life Sciences (DTL, http://www.dtls.nl/) and the FORCE11 (https://www.force11.org) or the Data FAIRport (http://datafairport.org/) initiatives. Data standards help to make data FAIR and contribute to the Open Access philosophy.

Furthermore, in the wake of recent scientific malpractice scandals, see (Fang et al. 2012), (Obokata et al. 2014), (Editorial 2014), (Stern et al. 2014) and news on the consequences,^1^ and in general the growing concern over the rise in paper retractions,^2^ governments and funding agencies are increasingly mandating reproducible research and the release^3^ and long-term archival of raw data with guaranteed rights to assess, review and appraise claims. Finally, the call for making publicly funded data be publicly available has resonated loudly and many groups are weighing-into end data retention by scientists^4^ (Molloy 2011).

The required infrastructure for open metabolomics data is getting into shape. The MetaboLights (Haug et al. 2013) repository at EMBL-EBI, for example, is experiencing a rapid growth and currently (as of July 2015) has about 165 complete metabolomics experiments, with about 53,000 samples and 1120 protocols captured. The cross-repository metabolomeXchange^5^ data-hub lists in total 270 (as of July 2015) publicly-accessible studies. Due to the submission and curation processes, these data sets are already standards-compliant at various levels.

One hurdle towards easy data access stems from the diversity of instrument vendor specific data formats. Working with these formats often involves commercial software or proprietary libraries, possibly with associated licensing costs and a restricted choice of operating systems. Such hurdles can rapidly impede access to data and limit seamless and efficient data flow in analysis pipelines. They also hamper the comparability of the results if data is to be processed by different vendor-specific software with possibly different algorithms. Such difficulties in data re-use are well known among bioinformaticians, and one of the main reason for standardisation efforts.

On one hand, it is fruitful to reduce the notational and semantic heterogeneity in experimental descriptions and results, to increase data interoperability and accelerate data integration. On the other hand, compliance with data standards is often perceived as an added burden. This is especially the case when data are produced and consumed locally in an insular manner, as compliance with the data standard requires extra—seemingly unnecessary efforts. However, considering the scientific enterprise as an increasingly interconnected activity, data exchange and preservation are both becoming essential requirements. Furthermore, national and international funding agencies are increasingly requesting publicly-funded research data to become *Open Access*.

But how are standards born in the first place? There are two main approaches: a “*bottom*-*up*” approach, usually by grass-root community efforts leading to an open (community agreed) standard, and a “*top*-*down*” approach, usually governed by a formal standardisation body. The eventual uptake and usage determines whether a specification becomes a “*de facto*” standard, or simply a “*de jure*” standard, which might be approved formally but not necessarily adopted widely. Most people working on such standards will understand the famous anecdote like “How Standards Proliferate” cartoon,^6^ describing a scenario where several standards already exist, but are found inadequate therefore yet another standard is proposed. This phenomenon can result in fragmentation among the developer- and user communities and cause friction resulting in an even lower adoption.

Standards are therefore social constructs and represent social agreements. To be successful, i.e. broadly adopted, the development needs to achieve a careful balancing act, ensuring both accurate description and ease of use. The Pareto rule could be the guiding principle, where the initial effort should cover 80 % of the use cases while the last 20 % would be the hardest to achieve.

In this manuscript, several areas where data standards are relevant in metabolomics will be covered. Examples will be given where standards succeeded, and “recipes” given on how to repeat such successes.

## Standards for vendor independent raw data in metabolomics

Excellent examples of how standards have evolved over time include the multiple data standards for mass spectrometry (MS) and NMR spectroscopy raw data, as described below, resulting in the widely used mzML format and emerging nmrML format.

### Mass spectrometry raw data standards

Early mass spectra were intended for human inspection, initially as images on photo plates, or printed as spectra or peak lists on paper. In the 1990s, the IUPAC CPEP Subcommittee on Electronic Data Standards developed the JCAMP formats^7^ for NMR and MS (Lampen et al. 1994) to harmonise the peak lists and associated spectral metadata in a human and computer readable manner. The human readability had disadvantages as the storage space for the textual representation required a whole byte for each digit. The Network Common Data Form (netCDF) was developed about 25 years ago (Rew and Davis 1990) for data in vector and array representations, such as geospatial data in climate models. The benefits of netCDF, which was optimised for efficient storage and access, lead to the specification of Analytical Data Interchange Protocol for Chromatographic Data^8^ or ANDI-MS for short (Erickson 2000), which was adopted by the American Society for Testing and Materials (ASTM).^9^

About 10 years ago, two separate XML standards were developed independently, mzXML (Pedrioli et al. 2004) under the guidance of the “mzXML-associated standard solutions” (MASS) Committee, and mzData (Orchard et al. 2004) within the proteomics standardisation initiative (PSI). By 2009, the best aspects of both mzXML and mzData were consolidated into a new standard called mzML (Martens et al. 2010) and resulted in joint support for a single open standard, thus eliminating duplicated efforts.

For all three XML based formats, the following factors were vital for broad adoption: (1) the support by vendors of MS instruments and the existence of freely available converters from vendor formats to the corresponding XML, (2) the availability of Open Source parser libraries, including validators to ensure completeness, consistency and unambiguous encoding of information. These in turn facilitate: (1) the broad support in Open Source research software and consequently (2) the adoption of mzML by major data repositories such as MetaboLights (Haug et al. 2013) and PRIDE (Jones et al. 2006), which both encourage or even enforce data deposition in vendor independent (non-proprietary) formats.

The mzML schema is generic enough to even support imaging mass spectrometry (Schramm et al. 2012). The imzML format includes the required controlled vocabulary and optimised data layout, but can be interconverted to “standard” mzML without information loss (Race et al. 2012). The optimized imzML is supported by both commercial and Open Source software, e.g. the Matlab-based MSiReader (Robichaud et al. 2013) or the R-Bioconductor based Cardinal package (Bemis et al. 2015).

There are remaining challenges for mzML and continued developments have been reported: for example, the mz5 format (Wilhelm et al. 2012) uses the same structure and all the ontology terms in mzML, but uses HDF5 as a container format, thus allowing full inter-conversion while benefitting from rapid access. Another improvement is the “numpress” compression scheme (Teleman et al. 2014) that allows a “lossy” representation of the binary spectral data, where the actual accuracy can be chosen at compression time.

**Fig. 1:**
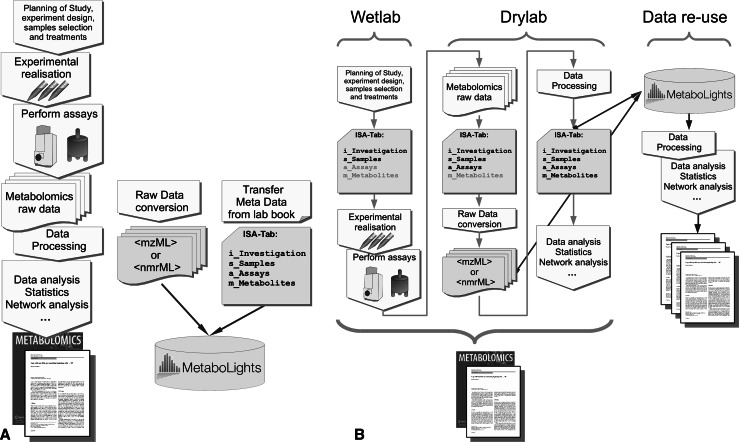
Experimental workflows in metabolomics. Shown in *light blue* are the relevant parts where data standards come into play. Annotated data deposition in open repositories allow for data re-analysis and re-use. **a** Traditional workflow using tools which do not depend on data standards, and where data annotation and data publication happen together with manuscript submission. **b** Fully standards embedded workflow, where data annotation is part of the standard operational procedures, data processing can use open software, and data publication is an integral part of the dissemination (Color figue online)

But what are the practical implications for the end users (biologists and analytical chemists) of a standard? At some stage, they need to convert MS raw data files from proprietary formats into an open format such as mzML. This will happen, either early and integrated with the experimental process, or only later nearer the time of (eventual) publication and data submission as shown in Fig. 1. An early conversion is necessary if vendor agnostic or open source data analysis tools are to be used. The reason that only a few open tools support proprietary formats is the added development effort and time required to enable import of these formats and keep them up to date. Usually, the vendors provide software libraries to access their own formats. The downside is that these often have rather complex application programming interfaces (APIs), and worse, each vendor has their own proprietary API. Currently, most of these interfaces require Windows dynamic link libraries (DLLs) for the actual file access, which are not compatible with other operating systems such as MacOSX or Linux.

The second reason to convert the vendor files is that the open formats can later be read by anyone, anywhere. Researchers can transfer data between institutions and collaborators, without the need for proprietary software (which might not be available at another location or laboratory). Another unwelcome but realistic scenario is that the software for older instrumentation is neither compatible with modern operating systems nor receives updates from the vendor for economic reasons. This is an extremely important aspect for long-term sustainability of data management in a research institution.

For these reasons, it is recommended to convert all files of a study to an open format soon after data collection, and retain them alongside the raw data in the original vendor format. One of the two main routes to mzML-formatted data is using Open Source converters such as the msconvert tool developed by the Proteowizard team (Chambers et al. 2012), which is one of the reference implementations for mzML. It can convert to mzML from Sciex, Bruker, Thermo, Agilent, Shimadzu, Waters and also the earlier file formats like mzData or mzXML and is consequently widely used. As the developers do not have access to all available instruments, support for the latest might take a while to implement, and in some cases the vendor-provided DLLs do not allow access to all features of the instrument. Although Proteowizard was initially targeting LC/MS data, it can also readily convert GC/MS data for example from the Waters GCT Premier or Agilent instruments. The other main route to mzML formatted data is by using vendor supplied converters where available, such as the Bruker CompassXport,^10^ AB SCIEX\MS Data Converter^11^ or in case of GC/MS for example the LECO ChromaTOF-HRT software. Only few vendor supplied converters are freely available and some require a commercial license. The wider community has to maintain constant pressure on all vendors to implement full access to our data in open formats. In the end, we are all their customers.

An important aspect is that metabolomics studies might comprise many raw data files, so the conversion from the vendor formats should not involve expensive manual intervention to add information beyond what is already stored in the instrument software. Furthermore, command line converters are easier to incorporate into local data processing pipelines. For bioinformaticians developing either software or databases, it is highly recommended to use existing I/O parsing software and libraries. Several such mzML libraries have been developed for different programming languages and software frameworks, summarised in Table 1.

**Table 1:** A selection of open source software libraries for reading, and for some writing, mzML

Language	Library/API	URL	License
Java	jmzML	https://code.google.com/p/jmzml/	Apache license 2.0
	jmzreader	https://code.google.com/p/jmzreader/	Apache license 2.0
C++	OpenMS	http://open-ms.sourceforge.net/	BSD
	Proteowizard	http://proteowizard.sourceforge.net/	Apache license 2.0
Python	pymzML	http://pymzml.github.io/	LGPL v3
R	mzR	http://bioconductor.org/packages/mzR/ https://github.com/sneumann/mzR	Artistic-2.0
MatLab	MSiReader	http://www4.ncsu.edu/~dcmuddim/msireader.html	BSD 3-clause
Ruby	mspire	https://github.com/princelab/mspire	MIT
Perl	MzML::Parser (CPAN)	https://github.com/Leprevost/MzML-Parser http://search.cpan.org/dist/MzML-Parser/	Dual: GPL or artistic license

See also http://www.ms-utils.org/wiki/pmwiki.php/Main/SoftwareList for a growing link of MS related software

### NMR raw data standards

For NMR data, The Metabolomics Innovation Centre (TMIC) in Canada and the COordination of Standards in MetabOlomicS (COSMOS) consortium (Salek et al. 2015) in Europe as well as other interested groups have developed the XML based, vendor-neutral open exchange and data storage format nmrML, which builds on efforts (Sansone et al. 2007) within the Metabolomics Standards Initiative (MSI) and work at the Wishart lab^12^ and earlier reporting requirements (Rubtsov et al. 2007). The format has also heavily borrowed ideas from the HUPO-PSI mzML standard (Martens et al. 2010), including an XML schema that defines the structure of an nmrML^13^ file and a supporting controlled vocabulary (nmrCV^14^), which allows the reuse of nmrCV terms in other formats and tools. The development of nmrML takes place on www.nmrml.org, where the specification documents, example files, and converters can be found. Java, Python, R and Matlab parsers have been developed to convert raw vendor formats to and from nmrML. Validator tools are available for quality control of the generated nmrML files, especially their completeness and correct semantics. The schema of nmrML has already been designed with 2D NMR experiments in mind, but the converters do not yet support 2D data. We would like to make developers of NMR data analysis software aware of our effort, and to welcome them to contact us and implement access to this open format. Likewise, users should start to consider submitting their 1D NMR data to metabolomics repositories such as MetaboLights (Haug et al. 2013) in the nmrML format.

## Study design and experimental metadata standards

We now discuss the differences between standards for instrument output and standards for experimental metadata and analysis reporting. The purpose of creating descriptive metadata is to facilitate discovery of relevant experimental data and to enable integrative and meta-analysis. The outcome of biological experiments is highly influenced not only by the experimental design or by the standard operating procedures used, but also by the many processing steps for peak picking, aligning, cleaning, transforming and the modelling of raw data. Therefore, to enable the precise reproduction of results, it is important to define reporting requirements associated with experimental design, data acquisition and variable manipulation during data processing and downstream statistical analysis. This is probably one of the most arduous tasks as the standardisation efforts need to be sufficiently generic to support a broad array of research questions and their particular experimental setup, but at the same time specific enough to ensure consistency, accuracy and reproducibility.

Several reporting guidelines have been created over the years, some of the first include the recommendations (Lindon et al. 2005) by the Standard Metabolic Reporting Structure (SMRS) initiative, a consortium of academic, government and industrial scientists which first met in 2003. Later, the Metabolomics Standards Initiative (MSI) was formed, and created a set of Core Information for Metabolomics Reporting (CIMR) guidelines, which were later published (Fiehn et al. 2007) as a set of articles in the *Metabolomics* journal.

### Formats for standardised metadata capture

More structured (digital) schemata have been proposed, including elaborate XML schema definitions (XSDs) or database models like ArMet (Jenkins et al. 2004) or SetupX (Scholz and Fiehn 2007), but also lightweight spreadsheet templates (Fernie et al. 2011). A nice summary of community accepted minimal information was presented in a recent editorial (Goodacre 2014).

Although the benefits of standard compliant reporting is undeniable, adoption is hampered by what is often viewed as a steep learning curve that can be time consuming for first time users. One remedy is to provide efficient software tools that integrate better with experimental workflows and provide configuration templates– sets of pre-defined attributes for different sample types used to capture metadata. Drop-down lists that limit the selection of particular fields would also improve software usability, as would improvements to available validation rules. However, it is just as important to provide appropriate training to scientists, to ensure they know how to perform and report reproducible research. Institutions increasingly have dedicated data managers who take care of the local data management infrastructure and can potentially provide such training.

The ISA-Tab format (Sansone et al. 2012) is a metadata standard that has gained a lot of momentum since its first release in 2008, and many of the reporting guidelines and considerations mentioned above have influenced its creation. The format comprises a set of tab delimited spreadsheet-like files that describe a given *Investigation*, including one or more *Studies* comprising a set of samples, and one or more *Assays* per study. The Investigation file captures the title, authors and a brief description of the underlying aim of a given investigation, a list of protocols applied, bibliographic information and contact data. Study files describe the origin of the sample material, its characteristics, protocols and experimental design factors relevant to the individual samples. Assay files specifically for metabolomics assays require information on how individual samples were extracted, possibly derivatized, and how the analytical protocols were performed for the actual measurements. For metabolomics, an additional fourth file type was specified by the developers of MetaboLights, which include tables of the intensities or concentrations of spectral features or metabolites in the samples. Depending on the platform technology, the table can be used to capture the metabolite-relevant analytical information such as chemical shift and multiplicity in NMR-based experiments, and m/z, retention index, fragmentation and charge for mass spectrometry. For identified spectral features, the metabolite information includes the name, external database identifiers, formula, and chemical structure as a SMILES or an InChI string.

ISAcreator (Rocca-Serra et al. 2010) is a standalone, Java-based, platform-independent desktop application with a range of facilities to enable standards-compliant creation of ISA-Tab archives. The software enables ontology searches and term lookup with a great deal of flexibility for capturing metadata at various stages of the experimental workflow.

Large portions of the data types, the actual Study layout, label descriptions, column names and recommended ontologies, are specified through a set of ISA configurations created with the ISAconfigurator. Several configurations exist for specific assay technologies, such as gene expression analysis, flow cytometry and different assay types in metabolomics. With these configurations, it is also possible to validate the metadata to ensure whether it complies with available ‘Metabolomics Standards Initiative’ (MSI) reporting recommendations. The ISAcreator metabolomics plugin developed at the EMBL-EBI captures the metabolites measured, with their quantification as described above.

As mentioned earlier, a factor that contributed to the widespread adoption of raw data standards was the support shown by vendors of MS instruments and the incorporation of the standards into their software. Similarly, incorporating the study design and experimental metadata standards into data processing and data management software promotes adoption of standards. The addition of standards into data management software, however, is not straightforward. This is because software such as Laboratory Information Management Systems (LIMS) and Electronic Lab Notebooks (ELN) are usually designed to be, and marketed as, generic products adaptable to a wide range of scenarios. Incorporating standards as part of these data management solutions attempts to make a generic solution work in a specific (standardised) way. However, with well-defined standards, this amalgamation should be achievable. Successful incorporation of standards into data processing and data management software would to some extent reduce the researcher’s manual data analysis efforts, thus yielding a tangible benefit for making data standards compliant earlier. Table 2 gives an overview of the software ecosystem around the ISA-Tab standard.

**Table 2:** Tools for customising, manipulating and processing ISA-Tab descriptions

Main functionality	Name	URL	Language/implementation	License
ISA-Tab configuration (creation of templates for ISA-Tab for specific domains)	ISAconfigurator	http://www.isa-tools.org/software-suite/, https://github.com/ISA-tools/ISAconfigurator	Java	Common Public Attribution License 1.0 (CPAL)
ISA-Tab creation and annotation	ISAcreator	http://www.isa-tools.org/software-suite/	Java	CPAL
	OntoMaton	https://chrome.google.com/webstore/detail/ontomaton/dkelbgmogiamnbbballckedaldbombni, https://github.com/ISA-tools/OntoMaton	Add-on for Google Spreadsheets	CPAL
ISA-Tab parser	PERL parser	https://github.com/bobular/Bio-Parser-ISATab	PERL	Dual: GPL or artistic
	Python parser	https://github.com/ISA-tools/biopy-isatab	Python	The MIT license (MIT)
ISA-Tab validation	ISAValidator	http://www.isa-tools.org/software-suite/, https://github.com/ISA-tools/ISAvalidator-ISAconverter-BIImanager	Java	CPAL
Browsing/visualisation of studies	BII web application	http://www.isa-tools.org/software-suite/	J2EE	MIT
	ISA-Tab Viewer	https://github.com/ISA-tools/ISATab-Viewer	Javascript	MIT
Conversion to other formats	ISAConverter	https://github.com/ISA-tools/ISAvalidator-ISAconverter-BIImanager	Java	Mozilla Public License (MPL) 1.1, GPL 2.0, LGPL 2.1
	isa2rdf	https://github.com/ToxBank/isa2rdf	Java	LGPL v3
	linkedISA	http://isa-tools.github.io/linkedISA/	Java	CPAL
Link to analysis platforms	Risa	http://bioconductor.org/packages/Risa/ https://github.com/ISA-tools/Risa	R, BioConductor package	LGPL
	GenomeSpace (online and through the ISAcreator tool)	http://www.genomespace.org and http://www.genomespace.org/support/guides/tool-guide/sections/isacreator	through ISAcreator, written in Java	LGPL
	Refinery	http://www.refinery-platform.org/, https://github.com/parklab/refinery-platform	Django/ Python	MIT-like Harvard license
	MetaDB	https://github.com/rmylonas/MetaDB	Grails/R	MIT and CPAL
XML-based experiment and metadata description tools	XEML-Lab	https://github.com/cbib/XEML-Lab	C++ (Windows, Mac and PC)	BSD
	Biocrates	http://www.biocrates.com/products/software	Windows	Commercial
	MASTR-MS	https://bitbucket.org/ccgmurdoch/mastr-ms/	Django/Python	GPL v3

Another approach is the development of interoperable tools, i.e., “metadata crosswalks” that facilitate exchange of metadata. A crosswalk is a data conversion that maps elements, semantics, or syntax from one metadata scheme to those of another. The degree to which these crosswalks are successful depends on the similarity of the two schemes, the granularity of the elements, and the compatibility of the content rules used to fill the elements of each scheme.

An example of such crosswalk in the case of metabolomics is the eXtensible Experiment Markup Language (XEML). The XEML-Lab (Hannemann et al. 2009) (https://github.com/cbib/XEML-Lab) is an XML-based framework for designing and documenting experiments in an intuitive yet machine readable format, and to link experimental metadata with any type of data generated in the corresponding experiments, and ultimately, to make both metadata and data available for data mining. XEML descriptions are used in both the Golm Metabolome database (Hummel et al. 2007; Kopka et al. 2005) (GMD, http://gmd.mpimp-golm.mpg.de) and the PLATO database (https://plato.codeplex.com) at INRA Bordeaux, which is a micro plate processing pipeline that supports enzyme activities and metabolite assays. The crosswalk is implemented in the XEML-Lab software, which can load experiments from these databases and export to ISA-Tab. If required, information that is missing can be added from within the XEML-Lab software. Other academic efforts also demonstrated the feasibility to export experimental data via metadata conversion to the ISA-Tab format as shown by the MASTR-MS LIMS solution.^15^, ^16^ Another example for the export of metabolomics data into standard formats is the very positive interaction with software vendors such as Biocrates AG (PRS, personal communication), showing that standard compliance does not have to be taxing for the users.

While such metadata crosswalks are essential, they are also labour intensive to develop and maintain. The mapping of schemes with fewer elements (less granularity) to those with more elements (more granularity) can be problematic.

## How to weave data standards into life-science experiments

Figure 1 shows two potential scenarios for standards compliant reporting of experiments. In Fig. 1a, the experiment is performed in the traditional manner from conception through to the manuscript writing. Journals are increasingly requiring that the underlying study data are made publicly available, so the relevant data and information are prepared for upload at the end of the process.

Getting familiar with the data management *life cycle* and tooling before starting a study can be very useful, since some kind of data organisation is always required. This moves data management from a retrospective activity to a prospective one. So making sure from the beginning that all information required later for publishing and data sharing is available in one place, rather than scattered across the hard drive and lab books, can be a time saver later. Standards need not be a hindrance, but should be perceived and understood as vehicles to increased trust, secondary usage and higher visibility of scientific output. Reused data is useful data and is data that gets cited (Piwowar et al. 2007). Standards compliance is just another standard operating procedure applied to the dissemination of the research output. This alternative approach is shown in Fig. 1b, where the whole experiment is *driven* by standards compliant results generation, here demonstrated using the ISA-Tab terms and concepts.

While it may sound trivial, creating a crisp title and short description of the Investigation as part of the ISA-Tab metadata helps focus on the question at hand. It is also beneficial if the institute or laboratory has established short guidelines on naming and the directory hierarchy. This helps to pass on institutional best practice to newcomers, just as for the laboratory SOPs. The ISA files can for example be kept close to experimental data, e.g. in the same directory.

Then, the Study table is populated with the sample details and the experimental design factors, such as genotypes, treatments or time points and very importantly, the tracking and annotation of QC samples. Often, such a table is used anyway using spreadsheet software to keep track of the samples. Furthermore, some MS or NMR instrument control software can use this information for the sample processing control, either directly or with small custom conversion scripts for each Assay.

Immediately after the measurements are performed, measured data should be converted to an open format such as mzML for both the subsequent processing and/or the later data publication, and the resulting filenames should be added to the Assay table. The ISA-Tab files now contain all information up to the data processing and analysis steps. Several data processing environments can take advantage of the annotation in ISA-Tab archives, for example the Galaxy workflow system (Goecks et al. 2010) and the R/Bioconductor framework (Gentleman et al. 2004). The R environment allows workflows to be written that combine the Risa (González-Beltrán et al. 2014) and xcms (Smith et al. 2006) packages, and the creation for example, of routine Quality Control reports for the whole experiment, or after further processing statistics and visualisations. The MetaDB (Franceschi et al. 2014) is a database and web application that provides a data processing workflow for untargeted MS-based metabolomics experiments with the incremental addition of ISA-Tab data as a core concept.

## On carrots and sticks, or “where there is a will, there is a way”

One of the hurdles on the road to standard adoption and uptake can be summarised in the question “What’s in it for me?” For an individual contributor, there can appear to be no immediate (short term) return on investment. A more top down solution is the creation and enforcement of data release policies which also include the recommendation to adopt data standards by funding bodies. The US NIH, for instance, imposes data release within 6 months of production. But data management is frequently regarded as the ugly duckling of bioinformatics, and the burden and costs of data management are often underestimated. Consequently the funding agencies, while mandating policies and recommending data standards, need to support data managers and research scientists for the extra expense in time associated with the additional work that standard compliance requires. Grant applications should thus include data management costs just like laboratory consumables.

On the bright side, publishers are playing an increasing role to reward scientists for their efforts in planning, producing and sharing datasets for the benefit of the scientific community. Datasets (and what are increasingly known as research objects) are being made citable and reusable, whose producers can be clearly identified, for instance by means of ORCID, which allows unambiguous tracking of persons and organisations. It has been shown that articles for which the data has been made available have increased citation rates (Piwowar et al. 2007). Nature Publishing Group’s *Scientific Data* and BiomedCentral’s *Gigascience* are what is known as ‘data journals’. These publications allow researchers to release their data and thereby provide the means for proper scholarly dissemination of their work via modern means, and without the need for a ground-breaking biological advance. This also has the added benefit of countering publication bias, where only positive results are published. Both journals support ISA-Tab format for structuring and releasing experimental metadata and issue DOIs for the data sets. Other journals such as *f1000Research* publish “Data Notes”, and more publishers are currently updating their data policies. Table 3 provides some examples for journal data deposition policies. A regularly updated list of journal research data policies is being compiled by the BioSharing Information Resource initiative^17^ in collaboration with a JISC pilot initiative.^18^ In BioSharing these will be cross-linked to the standards and databases, enabling access and cross-search of the information, on which a variety of stakeholders can base their decisions. Specifically, journals, researchers and funders will be able to recommend or select mature and community endorsed databases and standards, and developers and curators of repositories and content standards will be aware of the requirements they need to meet to ensure their products are discoverable and well described so that they can be used by researchers or recommended by journals and funders. Biosharing catalogue currently provides a dedicated collection, which lists standards and databases relevant to the field: https://biosharing.org/collection/MetabolomicsStandardsandDatabases.

**Table 3:** List of several journals publishing metabolomics research with strong data deposition policies as part of the respective instructions for authors

Journal	Policy	Journal link
Nature *Scientific Data*	Authors must deposit their data before submission, following the MSI guidelines. MetaboLights listed as recommended repository	http://www.nature.com/scientificdata/
*GigaScience*	Supporting data and source code must be publicly available, GigaScience provides the affiliated database GigaDB.	http://www.gigasciencejournal.com/
*Metabolomics*	It expected that data are made publicly available upon publication, suggestion to use MetaboLights or Metabolomics Workbench	http://link.springer.com/journal/11306
*Metabolites*	Authors are strongly encouraged to submit all supporting data to public, Open Access databases such as EMBL-EBI’s MetaboLights	http://www.mdpi.com/journal/metabolites
PLOS journals	All data underlying the findings described in a manuscript must be fully available	http://journals.plos.org/plosone/
f1000Research	Primary research articles should include the submission of the data underlying the results, together with details of any software used to process results […] Data are normally published under the CC0 licence which facilitates data reuse	http://f1000research.com/for-authors/data-guidelines

This is possibly a game changer as these initiatives provide a unique incentive for scientists to release their data in standard compliant fashion. In return? A higher visibility of the scientific output as data that can be trusted, mined, reused, mashed up and above all cited and acknowledged.

However, the metabolomics community lags 10 years behind the transcriptomics and proteomics communities in terms of learning-curve, maturity and acceptance of its resources. Metabolomics repositories face the same arduous situation as ArrayExpress (Brazma et al. 2003) or GEO (Edgar et al. 2002) when they were launched. MageML (Spellman et al. 2002) was the metadata scheme for transcriptomics experiments, but the lack of timely software support for this complex XML format led to the development of the simpler format MAGE-TAB. Many data standards in metabolomics such as ISA-Tab (Sansone et al. 2012), mzTab (Griss et al. 2014) and the mwTab used by the NIH metabolomics workbench have been modelled on, and learned from, the earlier -Omics formats. The combination of ‘arm twisting’ by publishers and funding agencies and at the same time loosening the annotation requirements resulted in the US and European repositories growing considerably. Today, no one doubts the value of these resources, as exemplified in several meta-studies (Chen et al. 2010), (Rhodes and Chinnaiyan 2005) and (Dhanasekaran et al. 2014). By now, data deposition to ArrayExpress and GEO is part of the routine work for anyone working on transcription profiling, and likewise the deposition of proteomics data to the member databases of the ProteomXchange consortium (Vizcano et al. 2014).

## Examples where data re-use boosted research

In metabolomics, as in other fields, the ability to download and use legacy data to demonstrate new or to compare existing data analysis approaches is where data standards and sharing excel. This was exemplified e.g. in (Gromski et al. 2014), where the authors used three different data sets from MetaboLights, including GC–MS and NMR datasets (MTBLS1, MTBLS24, MTBLS40) to investigate the effects of scaling metabolomics data prior to analysis with multivariate methods. The ability to use multiple data sets allows overall conclusions to be drawn on the most sensible scaling methods, which might then be generally applicable to similar metabolomics data.

Another example for re-use probably not anticipated by the original depositors is the MTBLS38 study in MetaboLights, which is a collection of biologically-relevant plant metabolite standards which were measured for the development and validation of MassCascade (Beisken et al. 2014). This data was used by M. Stravs (Eawag, CH), during a training workshop, to demonstrate the use of RMassBank (Stravs et al. 2013) to extract, annotate and recalibrate MS/MS data, and finally create 58 new reference spectra from MetaboLights (Haug et al. 2013) in MassBank (Horai et al. 2010).

The deposited data also helps in the development of novel computational approaches. Stanstrup and Vrhovšek used metabolite data from nine studies MTBLS4/17/19/20/36/38/39/4/52 and MTBLS87 along with other data sets for the development and evaluation of the www.predret.org retention time mapping database (Stanstrup et al. 2015).

In all these cases, the availability of the data in a standard format simplified or enabled the re-use. This demonstrated again that it is critical that publicly funded datasets are made available to the scientific community for mining and meta-analysis in a reasonable time frame.

An additional aspect pertains to the didactics of science: it will make training of data scientists easier, if real datasets can be used in textbooks and training courses. This requires trust: trust in the fact that repository content will grow and data will be discoverable; trust in the fact that enough individuals and institutes will contribute; trust that contributions will be of good enough quality so as to enable reuse, and trust that few will have their discoveries scooped. On this one last point, it seems that very few, if any, such cases can be documented. On the other hand, unrestricted access to data leads to critical review and early detection of reproducibility issues.

## Conclusion

Metabolomics standards have started to emerge about a decade ago, and this mostly concerned recommendations about which information had to be reported in the scientific literature. With increasing amounts of data being produced, mere description in manuscripts is no longer sufficient. We have shown that creating and sharing standards compliant data and metadata for metabolomics experiments is possible today.

At the same time, it is important to bear in mind that coming up with reporting guidelines is only one aspect of the standardisation process, and possibly even the easiest. The main challenge is to transform the guidelines into a robust syntax with defined semantics and to create successful reference implementations. These can only be achieved by building a set of free, vendor and platform independent software tools around the specifications for data manipulation, and to foster the buy-in from ‘power-users’ to ensure that relevant use cases are covered.

Most MS instrument vendors support raw data standards like mzML either directly or by collaborating with open source projects like Proteowizard. To be on the safe side, a tender description for a new instrument should include the requirement to export complete and fully calibrated raw data into mzML. If the analytics and data processing are outsourced, the contract should make sure that in addition to the results, also the primary and processed data are provided in open formats.

For metabolomics, metadata capturing has made big leaps in recent years. Not only have simple-to-process but versatile standards like ISA-Tab emerged, but tools such as ISAcreator have explored template generation for factorial study designs and this example should be followed for capturing experimental metadata. On top of that, metadata standards are increasingly used in data processing pipelines like MetaDB, or frameworks like R/Bioconductor and Galaxy, providing a carrot for users by simplifying the downstream data analysis steps.

By regularising how information is structured and reported, standards make it easier to distribute, disseminate and exchange information. Metabolomics repositories like the Metabolomics Workbench or MetaboLights are available to provide all data, and make it easy for scientists to fulfill the requirements of the journals to deposit research data associated with a manuscript. In related disciplines, annotation standards such as MIAME guidelines (Brazma et al. 2001) or the Gene Ontology (Ashburner et al. 2000) controlled vocabulary have become essential resources in modern molecular and computational biology.

Standards are developed to ensure that scientific information is delivered consistently, efficiently and meaningfully to the benefit of the community. Building such infrastructure does not occur overnight, and requires investment from all parties and also appreciation from funding agencies and stakeholders to acknowledge that data management is a new, essential scientific activity. This should be properly evaluated and factored in by funding agencies when supporting research efforts.

Therefore, instead of being seen as a burden, standardisation efforts and standards should be in fact perceived as unique helping tools to enhance the impact of the work carried out by scientists. Indeed, the examples presented above have shown that new types of research are made possible by exploiting a growing ‘data lake’, for example making it easier to assemble virtual cohorts by retrieving Open Access datasets for testing and evaluating algorithms or to perform meta-analysis.

Sometimes, it is simply about “just doing it”, or as the old adage goes, “where there is a will, there is a way”.

## Abbreviations

HDF5: Hierarchical Data Format, version 5
InChI: IUPAC International Chemical Identifier
ISA: Investigation Study Assay
IUPAC CPEP: International Union of Pure and Applied Chemistry, Committee on Printed and Electronic Publications
JCAMP: Joint Committee on Atomic and Molecular Physical Data
MASS: mzXML-associated standard solutions
netCDF: Network Common Data Format
PSI: Proteomics Standardisation Initiative
SMILES: Simplified molecular-input line-entry system
XML: eXtensible Markup Language

## Footnotes

1 “Japanese lab at centre of stem-cell scandal to be reformed…” 2014. 10 Mar. 2015 <http://blogs.nature.com/news/2014/08/japanese-lab-at-centre-of-stem-cell-scandal-to-be-reformed.html>.

2 “The Importance of Being Reproducible: Keith Baggerly tells…” 2013. 10 Mar. 2015 <http://retractionwatch.com/2011/05/04/the-importance-of-being-reproducible-keith-baggerly-tells-the-anil-potti-story/>.

3 “NIH Sharing Policies and Related Guidance on NIH-Funded…” 2007. 10 Mar. 2015 <http://grants.nih.gov/grants/sharing.htm>.

4 Free the Data Activity by Genetic Alliance <http://www.free-the-data.org/>.

5 http://metabolomexchange.org/.

6 http://xkcd.com/927/.

7 http://www.jcamp-dx.org/.

8 http://www.astm.org/DATABASE.CART/HISTORICAL/E1947-98.htm.

9 “ASTM International—Standards Worldwide.” 27 Mar. 2015 <http://www.astm.org/>.

10 “Software Downloads | Bruker Corporation.” 2012. 15 Feb. 2015 <http://www.bruker.com/service/support-upgrades/software-downloads.html>.

11 “Download—AB Sciex.” 2011. 10 Mar. 2015 <http://www.absciex.com/Documents/Downloads/Software/ABSCIEX-MS%20Data-Converter-User-Guide.pdf>.

12 http://www.metabolomicscentre.ca/exchangeformats.htm.

13 “nmrML—home.” 2012. 26 Mar. 2015 <http://nmrml.org/>.

14 http://nmrml.org/cv/.

15 "Mastr-ms code." 2013. 28 Mar. 2015 <https://bitbucket.org/ccgmurdoch/mastr-ms>.

16 "Mastr-MS — Mastr-MS 1.11.2 documentation." 2013. 18 Feb. 2015 <https://mastr-ms.readthedocs.org/https://mastr-ms.readthedocs.org/>

17 http://biosharing.org.

18 http://www.jisc.ac.uk/rd/projects/journal-research-data-policy-registry-pilot.
